# Defining Online Hating and Online Haters

**DOI:** 10.3389/fpsyg.2021.744614

**Published:** 2021-12-07

**Authors:** W. P. Malecki, Marta Kowal, Małgorzata Dobrowolska, Piotr Sorokowski

**Affiliations:** ^1^Faculty of Philology, Institute of Polish Philology, University of Wrocław, Wrocław, Poland; ^2^Institute of Psychology, University of Wrocław, Wrocław, Poland; ^3^International Center for Interdisciplinary Research, Silesian University of Technology, Gliwice, Poland

**Keywords:** online haters, trolling, cyberstalking, online hate speech, social media, online hate, attitudes, post-truth

## Abstract

According to a view widely held in the media and in public discourse more generally, online hating is a social problem on a global scale. However, thus far there has been little scientific literature on the subject, and, to our best knowledge, there is even no established scholarly definition of online hating and online haters in the first place. The purpose of this manuscript is to provide a new perspective on online hating by, first, distinguishing online hating from the phenomena it is often confused with, such as trolling, cyberstalking, and online hate speech, and, second, by proposing an operational definition of online hating and online haters based on ethnographic interviews and surveys of the existing scholarly literature.

## Introduction

According to a view widely held in the media and in public discourse more generally, online hating is a social problem on a global scale. It has been claimed to be at least partly responsible for the assassination of a well-known politician [Paweł Adamowicz, the mayor of one of Poland’s biggest cities (Nyczka, 2019)]; for various professionals having to flee their homes in fear for their safety (McDonald, 2014; Parkin, 2014), as well as for suicides among private individuals (Marcus, 2018). The negative influence of online hating seems to additionally extend even beyond those who are its direct target. Available data suggests that it is sufficient to merely witness online hating in order for one’s levels of subjective well-being to significantly decrease (Keipi et al., 2017). Finally, online hating seems to be an integral element of the general phenomenon of post-truth and fake news, much discussed in both scholarly literature and media outlets. Consisting in expressing unargued for negative assessments of others, online hating thrives in an environment where “public opinion is more influenced by fascinating emotions and subjective beliefs than by objective facts” (Scardigno and Mininni, 2020). However, thus far there has been little scientific literature on the subject (Lange, 2007), and, to our best knowledge, there is even no established scholarly definition of online hating and online haters in the first place. The purpose of this manuscript is to address this by proposing an operational definition of both online hating and online haters.

## Online Hating and Related Phenomena

One reason why there has been so little research dedicated specifically to online hating and online haters seems to be that online hating is often seen in academia as a mere variant of other online phenomena, most importantly trolling (March and Marrington, 2019), cyberstalking (Fearn, 2017), and online hate speech (Ortiz, 2019). This would explain the fact that while the public seems to see hating as a problem as serious as those three phenomena, the latter have been the subject of scholarly attention far more often than the former. In other words, to many scholars it is not yet clear that online hating is a separate phenomenon, so it is not yet clear whether a distinct definition is needed or even possible.

However, the evidence we have obtained suggests that the terms “hating” and “online hating” are often used to denote a phenomenon that is distinct from what is usually called “trolling,” “hate speech,” or “cyberstalking” – and, in fact, any other online phenomenon identified in scholarly literature – and which is at the same time of considerable social significance. So this phenomenon is definitely worth scholarly attention on its own. Our evidence comes from three sources: scholarly articles, media accounts, and ethnographic interviews.

We conducted systematic searches of both scholarly articles and media reports through the EBSCO Host database. We looked for scholarly articles/media reports published within the period of 2005–2021 that feature terms “online” + “haters” (33/154), “online” + “hating” (126/50), “online” + “hate” + “speech” (640/528), “online” + “trolling” (357/368), “online” + “trolls” (359/934), “cyberstalking” (406/134), and “cyberstalker” (15/14), with any duplicates being removed automatically by the system. Then we conducted a review of this material, taking into consideration additional articles and media reports on the above topics that we had known about from our previous work.

The ethnographic interviews were conducted specifically for the purpose of this study. The respondents to these interviews (*N* = 67) were graduate and undergraduate students at the University of Wrocław attending lectures given by one of the authors of the study. The interviews were conducted during classes, with the participants giving their responses anonymously on unsigned sheets of paper. For the sake of both time and anonymity, we abstained from asking demographic questions. However, the pool from which our sample was drawn allows us to estimate that most our participants must have been women and between 19 and 23 years old. Overall, our interviewees clearly recognized hating as a distinct phenomenon, were able to give its concrete examples, appeared to have witnessed it first-hand, and in some cases reported to have been its objects. Some of our participants admitted to having engaged in online hating in the past, expressing various attitudes toward these actions, ranging from regret to satisfaction.

In what follows, we will use data from these interviews, as well as the data we obtained from surveys of the existing scholarly literature and relevant media accounts, to spell out the differences between “online hating” “trolling,” “hate speech,” and “cyberstalking” by comparing online hating with each of these three phenomena. The purpose of this comparison, however, is not only to distinguish hating from those other phenomena, but also to help reach a definition. The comparison will be organized along the following three axes: *the purpose*, *the means*, and *the attitude*. For convenience, from now on we will use terms “online hating,” “hating,” “online hater,” and “hater” interchangeably. The *purpose* of online hating, first and foremost, is to publicly express a negative attitude toward a given person or object. As such, an act of hating is considered successful even if it provokes no reaction in others whatsoever. This clearly distinguishes hating from trolling, hate speech, and cyberstalking alike, all of which do aim at provoking certain reactions in other people. The purpose of trolling is to provoke a verbal reaction from the users of a certain platform – engaging them in a debate (Golf-Papez and Veer, 2017; March and Marrington, 2019). Hate speech aims to induce negative attitudes toward a given social group (a race, a gender, a nation, and so on) by expressing a disparaging opinion about that group (Nockleby, 2000; Ortiz, 2019). The purpose of cyberstalking is to harass – to cause discomfort to and hurt the interests of – a given individual, community, or a legal entity (Bocij and McFarlane, 2002; Fearn, 2017). Granted, hating may result in reactions such as those that hate speech, trolling, and cyberstalking aim at. Some haters may even relish those. But this does not change the fact that such outcomes are neither the primary intention of haters nor the primary purpose of hating. As our respondents put it: “Hating does not require causing any reaction, or discussion”; “Hating does not aim to initiate an argument, it does not require causing any reactions … although [haters] sometime do not shy away from arguments”; “Hating is just an intense expression of one’s feelings and thoughts.” The primary purpose of hating is achieved through the *means* of communicating verbal messages that carry a negative *attitude*. Among the characteristic examples of hating our subject gave there are: “Shitty song. You should never sing”; “Go and kill yourself.” Similar examples were given in an earlier study conducted in the United States: “This sucks. Go die” (Lange, 2007, 7). Most likely, it is this feature of hating that lies behind the custom of calling that phenomenon “hating” in the first place, referring to the common understanding of “hate” as “extreme dislike” (Merriam-Webster Dictionary Definition of HATE, 2021).

Although trolling, hate speech, and cyberstalking may all involve communicating such messages, doing so is not necessary for engaging in these behaviors. This is clear from the fact that the goals these behaviors aim at may be, and often are, achieved by messages that express a positive attitude or do not express any attitude at all. These may be, for instance, statements concerning a given group that are ostensibly positive or neutral (Cohen-Almagor, 2017) but also false in a way that hurts that person’s or group’s interests (hate speech and cyberstalking) or provokes a heated debate (trolling), or both. In addition to that, cyberstalking does not need to involve any verbal messages at all and is often achieved through such actions cybervandalism or identity theft (Lange, 2007; Sheridan and Grant, 2007). Finally, one feature that distinguishes hating from hate speech specifically, is that, unlike hate speech (Nockleby, 2000; Ortiz, 2019), hating does not necessarily consists in expressing a disparaging opinion about a social group and neither is it necessarily related to any political ideology. It may be disparaging without in any way referring to any ideology or the social identity of a given person or object and/or aiming at diminishing the social position of a group. As our subjects put it, hating may be purely “egoistical,” for instance, by embodying an attitude of “it is bad because I do not like it.”

## Defining Hating and Haters

Given the above, as well as other evidence we obtained through literature surveys and our ethnographic interviews, we might define online hating as the activity of posting online an explicitly negative assessment of a person or an object primarily for the purpose of expressing one’s negative attitude toward that person or object, independently of whether this will cause actual harm to a concrete person, provoke others to respond or whether it will diminish the value of a given social group. This purpose distinguishes hating not only from hate speech, trolling, and cyberstalking, but also from those forms of expressing negative attitudes such as critical reviews that aim to provide an informed opinion about a given person or object. Hating does not aim to provide an informed opinion but merely to express a negative attitude. This is why a typical manifestation of online hating is an explicitly negative assessment that is not argued for and therefore perceived as unconstructive. This defining feature of hating was stressed by participants in an earlier study on YouTube hating (Lange, 2007) and by our participants as well.

A hater is a person who routinely engages in hating behavior and it is reasonable to assume that such persons typically possess a common set of psychological features. It is also reasonable to assume that the characteristics of haters would be different than those common for people who engage in the other kinds of online behavior that were described above. While haters are likely to share some features with trolls, for instance, they are unlikely to share all of them. For instance, as both hating and trolling may result in upsetting people, these are unlikely to be engaged in by people with high or typical affective empathy. But at the same, while a troll will likely score high on cognitive empathy – without this he or she will not be able to accurately predict what will provoke people (Golf-Papez and Veer, 2017; March and Marrington, 2019; Moor and Anderson, 2019), this is not necessary for a hater. Similarly unnecessary for a hater are Machiavellianism, i.e., “a tendency to strategically manipulate others,” and narcissism, which are in turn typical for cyberbullies (Goodboy and Martin, 2015), and those engaging in hate speech (Withers et al., 2017), respectively.

Unfortunately, there is almost no research on the psychological features of haters, and the existing literature tells us only that haters are characterized by a low sense of self-identity, self-awareness, self-control (Chao and Tao, 2012), lack of confidence (Bishop, 2013), psychopathy (Sorokowski et al., 2020), high psychoticism mediated by cognitive distortion blaming others (Pace et al., 2021). The present research on hating (and its resulting definition of hating behavior) may anchor and provoke further studies, which could be based on the proposed systematization.

## Discussion

In this manuscript we have argued that there exists a distinct phenomenon of online hating and online haters that thus far has not been carefully discerned, and therefore studied, in scholarly literature. We would like to add to this now that while studying that phenomenon could yield results of scholarly significance, it is also difficult in methodological terms. The main difficulty here is related precisely to what, according to our interviews and literature review, distinguishes hating from the other forms of online harm that scholarly literature focuses on, that is, its intention. This is because that intention may often be difficult or impossible to deduce from a given utterance and the context that is accessible to the researcher. Some utterances, on their surface, may equally well qualify as hating, trolling, hate speech, cyberbullying, or some other form of discourse. But such cases should not discourage one from studying online hating. Firstly, such cases exist for any form of discourse that is defined in terms of intentions, including trolling and hate speech, yet many such forms, including trolling and hate speech, are studied despite of that. Secondly, one may give operational criteria that allow for qualifying an utterance as online hating based solely on their content, form, and the context that is easily available to researchers. If an utterance gives a negative assessment of a given person or object that is (a) not backed by any reasons, (b) does not appear controversial in a given environment, (b) does not have any explicit ideological content, then this is, most likely, an instance of hating.

In closing, we would like to argue that despite all the methodological, difficulties, online hating definitely deserves to be studied. This is not only because of scholarly but also practical reasons. After all, one might reasonably assume that online hating causes severe social harm, and that preventing that harm will not be possible without understanding online hating as such and implementing measures that are designed specifically with that phenomenon in mind.

## Data Availability Statement

The raw data supporting the conclusions of this article will be made available by the authors, without undue reservation.

## Ethics Statement

The studies involving human participants were reviewed and approved by the Research Ethics Committee at the University of Wrocław’s Institute of Psychology. The participants provided their written informed consent to participate in this study.

## Author Contributions

WM, PS, MK, and MD: conceptualization. WM: investigation and methodology. WM and MK: data management and analysis. WM, PS, MK, and MD: writing and editing. All authors contributed to the article and approved the submitted version.

## Conflict of Interest

The authors declare that the research was conducted in the absence of any commercial or financial relationships that could be construed as a potential conflict of interest.

## Publisher’s Note

All claims expressed in this article are solely those of the authors and do not necessarily represent those of their affiliated organizations, or those of the publisher, the editors and the reviewers. Any product that may be evaluated in this article, or claim that may be made by its manufacturer, is not guaranteed or endorsed by the publisher.
